# Keep in Touch with Buffalo

**Published:** 1904-07

**Authors:** 


### "Keep in Touch with Buffalo."

ON THE completion of their lecture courses it is customary for the professors of the medical department of the University of Buffalo to address the senior class, give the students advice of a general character, and wish them well in an informal way, in the careers they are about to enter upon. It marks the severance of relations between teacher and student and the words of kindly admonition usually sink deeply into the minds of the young physicians.

At the close of the last term of the medical department, one of the professors in the course of his remarks spoke of the work the University of Buffalo was doing and had been doing for more than half a century. He touched on the efforts of the successive faculties to bring the institution to the high state of efficiency it has attained, and predicted a greater future for the medical school which, beginning as an isolated little structure on the site of the old postoffice building, has grown to the magnificent proportions of the present High street group of University edifices. "But," he said, "the graduates must do their share of the work of making the University a really great place of learning. Don't forget the University," he said. "Don't go away and bury yourself. Keep in touch with Buffalo." That sentence is the key note of success for the University of Buffalo, and it applies to those who are residents of Buffalo as well as to those who have made their homes elsewhere. Keep in touch with Buffalo!

There are institutions of medical learning where the faculties are larger in numbers; where the list of instructors and lecturers presents an array of names covering page after page of the annual announcements; where every department of medicine and surgery is represented by a professor, clinical professors, instructors, clinical instructors, lecturers and demonstrators; but few institutions can better equip a man for the practice of medicine than the University of Buffalo. The chief difficulty with the University and the one serious bar to its becoming internationally known, save through the personality and reputations of such men as Park and Stockton and Mann and Cary, and their associates, is that it is not pushed. It has instead been conducted along the narrow lines of ethical standing. It has reached its present position after half a century of hard, unremitting work. The same position might have been reached in half the time with proper publicity. It receives students who present themselves; it has advertised its existence in a few medical journals and it sends out catalogues as does nearly every medical school. But it has lacked the advantages of an admissible amount of commercialism. Without endowments or other financial aid, battling through long decades of its existence, the faculties have given their best efforts, their time and all that was theirs to give for the advancement of the institution, and it stands today as a monument to their self-sacrifice in the cause of higher education,—one of the most carefully and scientifically conducted medical schools in the world. These men have taught medicine; they have not taught fads and fancies as have two internationally famous schools, one of which turns out notoriously arrogant and self-satisfied graduates and the other of which sends out good physicians but poor citizens, because they are saturated with antagonism to existing state laws regulating the licensing of practitioners of medicine.

The University of Buffalo has no fads; its aim and the endeavor of the men composing the faculty is to make good able men of its students; men who while they may not have the prestige of any one of the more exclusive institutions, are possessed of ability to properly diagnosticate disease conditions and intelligently and properly treat them. Geographically, it is admirably located; its clinical facilities are unsurpassed; its professors are eminent in scientific lines and in their ability to impart their knowledge to students. Then why is not the University of Buffalo one of the few really large instead of one of the many smaller medical colleges? Chiefly because we have not all kept in touch with Buffalo. Graduates have not shown the interest they should have shown in the institution after leaving it. On the other hand, has the faculty taken any steps to maintain interest in the college? Has the Council?

It will be conceded that the gentlemen at the head of the institution are very busy and have in spite of that fact devoted a great deal of their time to its advancement; they have done all that might properly be expected of them, and more, too, individually and collectively, but it might be productive of results if suggestions for the betterment of the college were invited, considered and acted upon.

It would be properly within the limits of permissible publicity to have the coöperation of an advisory committee made up of progressive men of business. Wisdom would seem to point to some of the newspaper proprietors as members of such a committee. Mr. George E. Matthews of the *Express*, has always been friendly to the University and its interests. Mr. E. H. Butler of the *News*, has not only been a friend of the University but has done much for it in a substantial manner. It was through the efforts of Mr. Butler and Dr. Park, seconded by Drs. Mann, Cary and Stockton, that the University secured the establishment of the cancer research laboratory, and it has been through the untiring efforts of these same gentlemen that the appropriation has been secured from year to year in spite of purely political opposition. Such a committee as this might well work independently of, but in thorough accord with, the members of the faculty and the council, and its secretary, in order that the bond of union between the bodies be closer, could be made assistant to the secretary of the faculty. Such an arrangement would be productive of nothing but good for the University of Buffalo and the efforts of the faculty and council to advance the interests of U. of B. would be materially aided. Of course, it is not necessary to here enumerate the methods by which university advancement would be accomplished; suffice it to say that results surely would be accomplished. The JOURNAL is committed to the advancement of the University and to that end has in course of preparation university material expecting it will be inaugurated in the fall as a part of the publication. But no matter what the JOURNAL may do individually as a publication, no matter how ceaselessly the faculty may labor as a body or as individuals, it will be uphill work and slow unless the alumni "keep in touch with Buffalo," and unless the outside world is placed in touch with the University of Buffalo and made familiar with it.

